# The relationship between safety net activities and hospital financial performance

**DOI:** 10.1186/1472-6963-10-15

**Published:** 2010-01-14

**Authors:** Jack Zwanziger, Nasreen Khan, Anil Bamezai

**Affiliations:** 1 Health Policy and Administration, School of Public Health, University of Illinois at Chicago, 1603 W Taylor St., Chicago, USA; 2 College of Pharmacy, 1 University of New Mexico, Albuquerque, NM, USA; 3 RAND Corporation, 1776 Main Street, Santa Monica, CA, USA

## Abstract

**Background:**

During the 1990's hospitals in the U.S were faced with cost containment charges, which may have disproportionately impacted hospitals that serve poor patients. The purposes of this paper are to study the impact of safety net activities on total profit margins and operating expenditures, and to trace these relationships over the 1990s for all U.S urban hospitals, controlling for hospital and market characteristics.

**Methods:**

The primary data source used for this analysis is the Annual Survey of Hospitals from the American Hospital Association and Medicare Hospital Cost Reports for years 1990-1999. Ordinary least square, hospital fixed effects, and two-stage least square analyses were performed for years 1990-1999. Logged total profit margin and operating expenditure were the dependent variables. The safety net activities are the socioeconomic status of the population in the hospital serving area, and Medicaid intensity. In some specifications, we also included uncompensated care burden.

**Results:**

We found little evidence of negative effects of safety net activities on total margin. However, hospitals serving a low socioeconomic population had lower expenditure raising concerns for the quality of the services provided.

**Conclusions:**

Despite potentially negative policy and market changes during the 1990s, safety net activities do not appear to have imperiled the survival of hospitals. There may, however, be concerns about the long-term quality of the services for hospitals serving low socioeconomic population.

## Background

The closing decade of the twentieth century witnessed dramatic changes in the US health care system; hospitals, accounting for the largest component of national health expenditures, were forced to respond to a series of measures designed to reduce health care costs [1]. The reductions in Medicare and Medicaid reimbursement under the federal Balanced Budget Act (BBA) of 1997 and the earlier growth of managed care organizations were both intended to reduce the growth of hospital revenue. Younis et al. (2005) examined the hospital's total profit margin before and after the BBA of 1997 [2]. Their results indicated that hospitals profitability decreased post BBA. Past research has indicated that during this period some hospitals were successful in maintaining their profitability; others experienced financial deterioration and, as a result, were sold or closed [3,4]. Studies of hospitals in New York City and California documented a widened gap between financially strong and financially weak hospitals [5,6]. The latter findings are of particular concern to policy makers since many of the financially weakest hospitals were those with significant burdens of "safety net" activities or that served low socioeconomic populations, Medicaid, and uninsured patients [7].

In addition to the changes in market and policy environments that affected all hospitals, there were changes that specifically impacted hospitals that were disproportionate providers of safety net activities or hospitals that serve vulnerable populations such as the poor, Medicaid beneficiaries and the uninsured [8]. Potential impacts included an increase in the number of uninsured, increased competition for the lowest-risk Medicaid patients enrolled in Medicaid managed care plans, increased price-shopping by private insurers, increased concentration of uncompensated care in a smaller number of hospitals, and the slowdown in the growth in Medicaid payment rates [9-15]. For example, Gaskin et al. (2001) found that safety net hospitals were losing low risk Medicaid patients as hospitals market became more competitive over time. Gruber (1994) and Zwanziger et al. (2000) found that price competition from managed care plans reduced hospitals' ability to increase rates for privately insured patients to compensate for their losses from treating uninsured or under-insured patients. Dranove et al. (1998) reported that Medicaid-dependent hospitals were not able to cost shift to other paying populations and, when faced with budgetary constraints, responded by decreasing the number or quality of services provided; such hospitals were also more likely to close. This threatening combination of policy and market changes suggests that hospitals providing disproportionate share of safety net activities faced sharply increased fiscal pressures during the 1990s, though only limited direct evidence exists to support this conclusion.

Empirical studies of hospitals with high safety net activities have produced no clear consensus as to their financial status. Both Zuckerman et al. (2001) and Bazzoli et al.(2005) for example, found that hospitals ranked in the highest 10th percentile in the provision of uncompensated care and/or of hospitals with correspondingly disproportionately high shares of uncompensated care performed more poorly compared to the profitability of other hospitals during 1990-2002 [16,17]. Other studies found that hospitals that served vulnerable populations were able to survive during 1990s by adopting strategies that decreased costs and maximized revenues in response to market and policy changes [7,18]. For example, Felland et al. (2003) found that safety net hospitals in 9 of 12 communities studied were not only intact from 1990-2000 but were also able to expand and improve services to the uninsured by streamlining their operations, engaging in integration, and actively pursuing paying patients. Other studies found no, or even positive, relationships between safety net activities (such as providing care to the uninsured and Medicaid beneficiaries) and financial performance [19-22]. However, majority of these studies were focused on specific geographical areas limiting the generalizability of these studies to respond to concerns about the continued financial health of hospitals that provide services to vulnerable populations.

The primary objective of this paper is to use data from the 1990s to trace the relationship between safety net activities and two dimensions of financial performance-profitability and operating expenditure, among all urban hospitals. We chose those dimensions because they provide complementary perspectives on the financial impacts of safety net activities during this period of market and policy turbulence. Profitability provides an indicator of a hospital's long-term viability since chronically unprofitable hospitals tend to close [23]. But maintaining profitable margins is not enough from a policy perspective, particularly if hospital accomplishes this at the expense of quality [10,24]. One example of undesirable means of maintaining profitability would be to lower expenditures by reducing the number of services or mix of care provided to low income and/or uninsured population. For instance, Langa and Sussman (1993) found that hospitals reduced the intensity of services provided to Medicaid patients in response to financial cuts [25].

We assess the relationship between two safety net activities, the provision of care to Medicaid beneficiaries and to low socioeconomic populations, and their effect on total profit margin and operating expenditure. In addition, we take advantage of the availability of uncompensated care data from 2002 and test the effect of adding this safety net activity to our analysis. We use multivariate regression models with controls for many time-varying measures and hospital fixed effect estimation approach, which controls for hospital specific time-invariant measures. By using large, nationally representative, longitudinal data, and a robust research design we have improved on earlier studies and provide credible estimates of the relationship between safety net activities and hospitals financial performance.

## Methods

### Data

We focused our analysis on urban hospitals, defined as those located in any metropolitan statistical area (MSA). The sample was further limited to nonfederal, short-term, general acute care hospitals-hospitals most accessed by patients from vulnerable populations. The period studied was 1990-1999-a time when the hospitals faced major policy and market pressures. Our national, hospital-level database combined information from several sources. Data on hospital annual operating revenues, expenditures, discharges, teaching intensity, and payer mix were obtained from the annual Medicare Hospital Cost Reports spanning the entire decade. Reporting cycles varied between hospitals, so data from successive cost reports were linked and then annualized. These data were supplemented by additional hospital characteristics such as total outpatient visits and hospital ownership from the American Hospital Association Annual Hospital Survey (1990-1999). The Medicare Hospital Market Service Area Files (HMAF) for 1989, 1995, and 2001 were used to define hospitals service areas. The socioeconomic characteristics for each service area were calculated using 1990 and 2000 US Census of Population and Housing data and were estimated for 1995. Finally, we used uncompensated care charges available in the 2002-2003 CMS-2552-96 Hospital Cost Report files to calculate each hospital's uncompensated care burden.

The level of competition was measured through Hirschman-Herfindal indices (HHI) (defined below) for years 1990, 1995, and 2000 and imputed for intermediate years. The hospital HHI used the Medicare HMAF data and data on system membership from the Williamson Institute multi-hospital system for years 1989, 1995 and 2001. The mean health maintenance organization (HMO) penetration by provider was generated using the InterStudy Regional Market Analysis database for 1994-1999. HMO penetration was defined as the proportion of patients in a geographic region enrolled in any HMO in the hospital market.

There were 24170 hospital*year observations in the AHA data. Of these, 7490 observations were deleted due to missing financial data. The mean differences on hospital characteristics between omitted and included observations, although statistically significant, were small. The hospitals that were omitted were slightly smaller and for-profit. The final sample size was 16,680 hospital*year observations.

### Construction of dependent variables and covariates

#### Dependent variables

We used the ratio of total hospital revenue to total expenses-rather than the corresponding operating measures [22] - to measure profitability since we want to assess the impact of safety net activities on financial viability where the effect of non-operating revenue, e.g., public subsidies, are critical. As hospitals might maintain profit margins by decreasing expenses (and thereby quality), our other dependent variable was operating expenditures. We used logged forms to account for skewness associated with such measures.

#### Safety net activities

We focused on two measures of safety net activity: serving a low socioeconomic population and Medicaid intensity. These measures were selected based on IOM definition which defines "core safety net hospitals" as those serving uninsured, Medicaid and other vulnerable population For a more detailed description of the construction of the safety net activities readers are referred to [8].

The safety net variables were constructed as follows:

##### a) Serving a low SES population

The socioeconomic status (SES) of the patients residing in a hospital service area was calculated based on the hospital's discharges. We first identified a hospital's service area based on the zip codes that cumulatively accounted for 75% of its discharges, using the Medicare HMAF files. We then used the census data to estimate four SES measures for these zip codes-weighting by the size of the relevant population in the zip code area: a) the percentage of the population 25 years of age and older that did not have high school diplomas, b) the percentage of minority residents (African-American, Native American, and non-black Hispanic), c) median household income, and d) percentage of the residents with incomes below the poverty line. As the SES measures were highly correlated, we extracted a common measure using principal component analysis for each study year. This common measure was defined as the "SES index." The index was normalized annually to have a mean of zero and a variance of one. Higher values of the index indicate lower socioeconomic status.

##### b) Medicaid intensity

Medicaid intensity was the proportion of a hospital's admissions that were insured by Medicaid, adjusted for overall average proportion of Medicaid admissions in the MSA as we wanted to focus on hospital relative participation in safety net activities within MSAs.

In some specifications, we added uncompensated care burden. The uncompensated care burden was calculated from the uncompensated care charges divided by total charges extracted from the Medicare Cost Report data set.

#### Market competition

We used the Medicare HMAF data to calculate the competitiveness of each hospital's market. The Medicare HMAF data sets provided the number of Medicare patients discharged from a hospital that reside in a given zip code area (ZCA) in a given year. The measure of competition we used is HHI, a measure of competition developed by economists to incorporate both the number of competitors and their relative market share in a single measure[26,27] is defined as follows:

where the sum is over all the competitors in the market.

We found the market shares of the competing hospitals in each ZCA and then summed the squares of these market shares to calculate the ZCA's HHI. We then calculated the relative proportion of each hospital's discharges contributed by each ZCA. These proportions were used to calculate a weighted average HHI as the measure of the competitiveness of the hospital's market. The following formula summarizes the calculation:

where

HHI_i_-is the HHI for the i^th ^hospital; w_ij_-is the proportion of discharges from hospital i that reside in ZCA j; HHI_j_-is the HHI for the j^th ^ZCA.

The specific algorithms used are described elsewhere [28,29]. The HHI calculated in this manner assume that the hospitals are all independent. We then incorporated membership in local hospital systems by changing the calculation of the ZCA HHI. The specific algorithms are described elsewhere [30]. Membership information was obtained from the Williamson Institute of the Medical College of Virginia provided the lists of member hospitals and systems for 1989, 1995, and 2001 using a variety of sources including the American Hospital Association's Annual Survey of Hospitals, the Institute's own surveys, and direct contacts. Systems were only based on common ownership within the same MSA and did not include looser, contractual affiliations.

#### Other covariates

We used the Medicare wage index from the Centers for Medicare and Medicaid Services (CMS) to control for differential geographic labor input prices. The models also include the hospital case mix index from the CMS to account for severity of the discharges across the hospitals. We also included input price measures such as discharges, visits, and Medicare patient mix. Further, all model specifications control for year fixed effects.

#### Instruments for Medicaid

We used an instrumental variable approach to account for potential endogeneity of the Medicaid intensity. We identified determinants of Medicaid intensity from the literature that were plausibly uncorrelated with dependent variable conditional on covariates. We experimented with the following instruments: MSA-level employer characteristics from Statistics of US business; number of public hospitals in the county and presence of a trauma center from AHA; MSA population size and proportion of Hispanics and African-Americans from Area Resource File; unemployment rate from Bureau of Labor Statistics; and Medicaid eligibility thresholds for pregnant women, children, and elderly collected from published resources [31-34]. Based on the results from the first stage and the over-identification test, the best set of instruments were state-level Medicaid eligibility thresholds for elderly, medically needy thresholds for elderly and county-level unemployment rate.

### Model specification

Hospital profitability is broadly determined by the relationship of the demand for a hospital's services to the costs of the inputs used to produce them. The demand for a hospital's services will depend on its characteristics (ownership, size and teaching status), the population it serves (demographics including their socioeconomic characteristics and health status), and its market environment (particularly managed care penetration and the competitiveness of the hospital's market). Hospital cost structures largely depend on: input costs, particularly wage rates; outputs (reflecting the economies or diseconomies of scale and scope); and market and payer environments [28,35]. We test whether hospitals' safety net activities influence hospitals profitability and/or hospital expenses. Such relationships are plausible since safety net activities could both increase cost (e.g., due to a more acute case mix, increased wage rates due to higher demand for services). The situation may have been exacerbated during 1990s when hospitals were faced with potentially disadvantageous policy and market changes.

In developing the models, we follow the methodology specified in Zwanziger et al. (2000) [14]. We used the logarithm of the output and input price levels and hospital-specific fixed effects estimator in a multivariable regression model. The hospital-specific fixed effects specification reduces the impact of potential omitted variables that are relatively stable over time and hospital-specific. By focusing on the relationship between changes in each independent variable and profitability over time, the fixed effects specification provides a more robust indicator of relationships between dependent and independent variables than does Ordinary Least Squares (OLS). We tested whether the fixed effects were significant and if random effects would be more appropriate. The fixed effects specification was significant at p = 0.01. Further, the Hausman specification test found systematic differences between fixed and random effects coefficients indicating that fixed effects would be an appropriate choice. Finally, in view of the potential endogeneity of Medicaid intensity, we used instrumental variable estimation approach. Medicaid intensity can be endogenous because higher Medicaid admission can possibly tend to lower profitability (due to lower reimbursement rates) but, simultaneously, lower profitability may possibly lead to higher proportion of Medicaid patients (e.g., due to a fewer amenities). We tested for the validity of the instruments using first-stage, over-identification, and endogeneity tests [36,37].

For all of these specifications, we used two versions of the safety net activities: a continuous version that tested for an overall relationship between the intensity of the activity and the dependent variable of interest and an indicator variable for the highest 5th percentile for each measure to test for possible threshold effects. We used the top 5th percentile cutoff because hospitals in the top 5th percentile were most likely to be stable on safety net activities over time (results not shown).

The models estimated had the following variables:

where

Yit is the log total margin (or operating expenditure) of hospital i in year t,

• OUTPit are outputs (discharges, outpatient visits, and second order interactions) of hospital i in year t,

• TEACHit is a measure of teaching activities (intern/bed ratio) of hospital i in year t,

• MANi is the mean HMO penetration in hospital i's market,

• COMPit is the concentration (HHI adjusted for membership in local hospital systems) for hospital i in year t. For this variable t takes on the values, 1990, 1995, and 1999,

• MEDit is the proportion of Medicare discharges for hospital i in year t,

• SNit are the safety net activities (SES index, Medicaid intensity, uncompensated care burden) for hospital i in year t,

• CMit is the case mix index of hospital i in year t,

• MWit is the Medicare wage index of hospital i in year t,

• YEAR t is the year indicator variable for year t, and

• INTER it are the interactions of market and safety net activities with year dummies for hospital i in year t

• fi is the hospital fixed effect, and

• eit is the error that is i.i.d.

Safety net activities, payer mix, HHI and managed care penetration are interacted with time to allow for differential time effects.

## Results

Table 1 present the means and standard deviations of the variables in the multivariate models. The average total margin for the hospitals studied during the period was 5%. The average Medicaid intensity was 14%.

**Figure 1:**
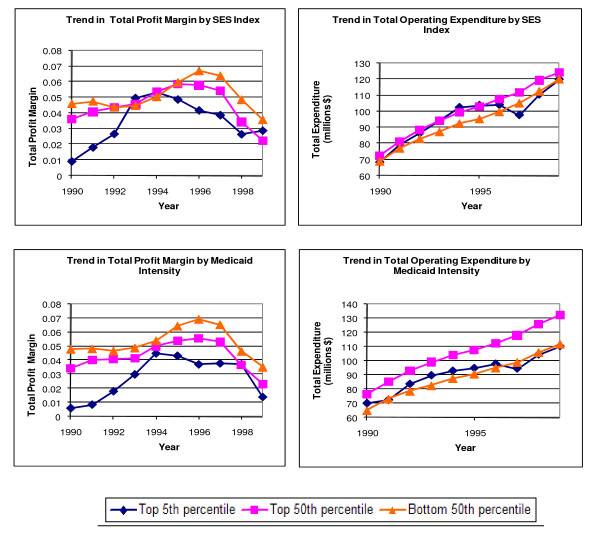
**Trend in total profit margin and operating expenditure by safety net activities**.

Figure 1 provides comparisons of total profit margins, and operating expenditures during the 1990s by the intensity of their safety net activities. Figure 1 does not adjust for any covariates. In general, the profit margin increased in the early 1990s reaching their peak in 1996 and dropping thereafter. This drop is consistent with the drop in hospitals profit during 1997 following the introduction of the BBA of 1997. For hospitals in the highest 5^th ^percentile of safety net activity, peak profitability was in 1994. Operating expenditures continued to increase in the 1990s, dropping in 1997 for hospitals with the highest 5^th ^percentile safety net activities and increased thereafter. In general, all the graphs display a consistent inverse relationship between profitability and safety net activities providing preliminary support for the view that hospitals with disproportionately higher safety net activities tended to be under greater financial stress than hospitals with lower safety net activities. However, it is not clear whether safety net hospitals were particularly disadvantaged during the study period.

**Table 1:** Means and standard deviations of variables in the regression model

Variable	Mean	Std. Dev.
Total operating margin	0.0003	0.0817
Total profit margin	0.0480	0.0690
Total expenditure (millions $)	96.900	92.000
SES index^a^	-0.0993	0.9252
Medicaid intensity	0.1375	0.1218
Uncompensated care burden	3.7013	5.3715
Discharges	10938	7887.0
Outpatient visits	122389	116644
Intern-to-bed ratio	0.0549	0.1267
Not-for-profit	0.7363	0.4407
For-profit	0.1436	0.3507
System HHI	0.3642	0.1175
Mean HMO penetration	0.2565	0.1374
% Medicare admission	0.4801	0.1379

N	16680	

**Notes**:

Source: AHA Annual Hospital Survey and Medicare Hospital Cost Reports (1990-1999)

^a^A high score on SES index indicates low socioeconomic status

Table 2 displays the estimated coefficients of the effect of safety net activity on profitability using OLS and hospital fixed effects specifications for continuous safety net activities and fixed effects specification for binary forms. The coefficients of Medicaid intensity were not statistically significant in either OLS or fixed effects specification except for 1997. In 1997, this coefficient was positive and marginally significant in the fixed effects model indicating that Medicaid intensity was more profitable for that year. The coefficients of the SES index from the OLS model were significantly negative until 1991, rose in the mid-1990s, and were significantly negative in the later years. The fixed effects model, however, indicated that the SES index was not significantly different from zero and the coefficients were small.

**Table 2:** Effect of safety net activities on hospital total profit margin

	Continuous safety net measures		Binary safety net measures
	
	(1) OLS	(2) Fe	(3) Fe-Binary
Log Medicaid intensity * 1990	-0.037 [0.023]	-0.021 [0.027]	
Log Medicaid intensity * 1991	-0.040 [0.022]	-0.025 [0.024]	-0.005 [0.007]
Log Medicaid intensity * 1992	-0.016 [0.023]	-0.002 [0.022]	0.011 [0.009]
Log Medicaid intensity * 1993	-0.034 [0.020]	-0.016 [0.021]	0.015 [0.010]
Log Medicaid intensity * 1994	-0.012 [0.019]	0.032 [0.020]	0.024 [0.010]*
Log Medicaid intensity * 1995	-0.017 [0.021]	0.011 [0.019]	0.024 [0.011]*
Log Medicaid intensity * 1996	-0.013 [0.023]	0.033 [0.021]	0.026 [0.010]*
Log Medicaid intensity * 1997	0.004 [0.025]	0.057 [0.023]*	0.033 [0.011]**
Log Medicaid intensity * 1998	0.016 [0.030]	0.046 [0.028]	0.048 [0.015]**
Log Medicaid intensity * 1999	0.001 [0.028]	0.030 [0.028]	0.029 [0.013]*
SES index^a ^*1990	-0.007 [0.002]**	0.003 [0.007]	
SES index *1991	-0.005 [0.002]*	0.006 [0.006]	0.006 [0.007]
SES index *1992	-0.001 [0.002]	0.009 [0.006]	0.014 [0.011]
SES index *1993	0.002 [0.002]	0.012 [0.006]	0.032 [0.011]**
SES index *1994	0.001 [0.002]	0.011 [0.007]	0.026 [0.012]*
SES index *1995	-0.001 [0.002]	0.009 [0.007]	0.018 [0.012]
SES index *1996	-0.007 [0.002]**	0.003 [0.006]	0.009 [0.012]
SES index *1997	-0.008 [0.003]**	0.001 [0.007]	0.010 [0.013]
SES index *1998	-0.012 [0.003]**	-0.001 [0.007]	0.004 [0.015]
SES index *1999	-0.010 [0.003]**	0.001 [0.007]	0.028 [0.017]
log(case mix index)	-0.365 [0.119]**	0.020 [0.156]	-0.010 [0.156]
log(discharges)	0.182 [0.037]**	0.334 [0.065]**	0.345 [0.065]**
Log(visits)	0.003 [0.026]	0.013 [0.027]	0.008 [0.028]
Log(discharges)squared	-0.008 [0.003]**	-0.017 [0.004]**	-0.018 [0.004]**
Log(visits)squared	0.001 [0.002]	-0.001 [0.001]	-0.001 [0.001]
Log(case mix)*log(discharged)	0.044 [0.013]**	-0.001 [0.017]	0.003 [0.017]
Log(visits)*log(discharges)	-0.002 [0.003]	0.002 [0.003]	0.001 [0.003]
Log(Medicare wage index)	0.060 [0.092]	0.307 [0.167]	0.282 [0.166]
Log(Medicare wage index)squared	-0.125 [0.034]**	-0.044 [0.042]	-0.040 [0.042]
Log(visits)*log(Medicare wage index)	0.005 [0.010]	-0.010 [0.011]	-0.008 [0.011]
Log(discharges)*log(Medicare wage index)	-0.016 [0.012]	-0.014 [0.019]	-0.013 [0.018]
Log(intern-to-bed ratio)	-0.063 [0.012]**	-0.103 [0.042]*	-0.103 [0.042]*
Not-for-profit	-0.007 [0.003]*		
For-profit	0.020 [0.005]**		
1991 dummy	0.004 [0.012]	0.004 [0.010]	0.003 [0.008]
1992 dummy	-0.010 [0.013]	-0.004 [0.013]	-0.003 [0.010]
1993 dummy	-0.031 [0.013]*	-0.027 [0.013]*	-0.031 [0.010]**
1994 dummy	-0.021 [0.015]	-0.025 [0.014]	-0.016 [0.011]
1995 dummy	-0.013 [0.015]	-0.010 [0.015]	-0.009 [0.011]
1996 dummy	-0.018 [0.016]	-0.020 [0.016]	-0.011 [0.012]
1997 dummy	-0.020 [0.017]	-0.020 [0.016]	-0.006 [0.012]
1998 dummy	-0.035 [0.018]	-0.020 [0.018]	-0.013 [0.014]
1999 dummy	-0.044 [0.019]*	-0.027 [0.019]	-0.022 [0.015]
Log percent Medicare*1990	-0.063 [0.023]**	0.002 [0.032]	-0.007 [0.030]
Log percent Medicare*1991	-0.065 [0.019]**	-0.002 [0.030]	-0.013 [0.028]
Log percent Medicare*1992	-0.035 [0.023]	0.018 [0.029]	0.008 [0.028]
Log percent Medicare*1993	-0.013 [0.020]	0.047 [0.028]	0.049 [0.027]
Log percent Medicare*1994	-0.029 [0.019]	0.038 [0.026]	0.019 [0.025]
Log percent Medicare*1995	-0.003 [0.021]	0.057 [0.026]*	0.050 [0.025]*
Log percent Medicare*1996	0.010 [0.023]	0.075 [0.027]**	0.061 [0.025]*
Log percent Medicare*1997	0.011 [0.023]	0.075 [0.027]**	0.056 [0.025]*
Log percent Medicare*1998	-0.020 [0.026]	0.018 [0.030]	0.017 [0.028]
Log percent Medicare*1999	-0.020 [0.026]	0.014 [0.030]	0.013 [0.029]
Log(System HHI)*1990	0.025 [0.004]**	0.003 [0.011]	0.001 [0.010]
Log(System HHI)*1991	0.025 [0.004]**	-0.000 [0.011]	-0.004 [0.011]
Log(System HHI)*1992	0.026 [0.005]**	0.002 [0.011]	-0.003 [0.011]
Log(System HHI)*1993	0.014 [0.004]**	-0.008 [0.011]	-0.010 [0.011]
Log(System HHI)*1994	0.010 [0.004]*	-0.013 [0.011]	-0.016 [0.011]
Log(System HHI)*1995	0.020 [0.005]**	-0.002 [0.011]	-0.005 [0.011]
Log(System HHI)*1996	0.019 [0.005]**	-0.002 [0.011]	-0.002 [0.011]
Log(System HHI)*1997	0.025 [0.006]**	0.003 [0.012]	0.004 [0.012]
Log(System HHI)*1998	0.018 [0.008]*	-0.001 [0.013]	0.004 [0.013]
Log(System HHI)*1999	0.022 [0.007]**	0.006 [0.012]	0.012 [0.013]
Mean HMO penetration*1990	-0.039 [0.011]**		
Mean HMO penetration*1991	-0.040 [0.010]**	-0.006 [0.009]	-0.012 [0.008]
Mean HMO penetration*1992	-0.038 [0.011]**	-0.005 [0.012]	-0.018 [0.012]
Mean HMO penetration*1993	-0.023 [0.011]*	0.013 [0.013]	-0.003 [0.012]
Mean HMO penetration*1994	-0.038 [0.011]**	0.000 [0.013]	-0.014 [0.012]
Mean HMO penetration*1995	-0.044 [0.011]**	-0.009 [0.014]	-0.019 [0.013]
Mean HMO penetration*1996	-0.040 [0.012]**	0.001 [0.015]	0.001 [0.014]
Mean HMO penetration*1997	-0.040 [0.013]**	-0.001 [0.015]	0.001 [0.014]
Mean HMO penetration*1998	-0.040 [0.016]*	0.006 [0.017]	0.012 [0.016]
Mean HMO penetration*1999	-0.030 [0.017]	0.025 [0.019]	0.032 [0.018]
Observations	16680	16680	16680

Notes:

Source: AHA Annual Hospital Survey and Medicare Hospital Cost Reports (1990-1999)

^a^A high score on SES index indicates low socioeconomic status Robust standard errors in brackets

*Significant at p < 0.05; ** significant at p < 0.01%

Table 2 also presents fixed effect specification for binary safety net variables. The models trace time interaction for safety net activities with 1990 as the reference year. The coefficient on Medicaid intensity is positive and significant beginning in 1994, indicating that hospitals with high Medicaid intensity were more likely to be profitable in later years relative to 1990. For the SES index the time interactions were positive and significant for 1992 and 1993, indicating that hospitals with low SES population had higher profits for those years relative to 1990.

We also estimated the models using 2SLS to account for potential endogeneity of Medicaid intensity. The partial F test for our excluded instruments from the first stage (for Medicaid intensity) was F (2, 1725) = 82.45. The Hansen J statistics for the over-identification test for the instrument had p value of 0.1536, suggesting that our instruments were not correlated with profit margin. Hence, the two tests indicate that our instruments predicted Medicaid intensity and are valid. In addition, we tested for the endogeneity of our Medicaid intensity using a Hausman-Specification test. The *P *value for this test was 0.9269; thus, we cannot reject the null hypothesis of exogeneity of Medicaid intensity. This was also evident when we compared the estimates of Medicaid intensity between OLS and fixed effects. The estimates are identical across the two specifications, bolstering the possibility that Medicaid intensity in our sample is exogenous.

Results from models that included uncompensated care burden were similar to the models without uncompensated care (data not shown). Inclusion of uncompensated care did not influence the coefficient of other safety net activities. In general, we did not find any statistically significant impact of uncompensated care burden on profitability and its time interactions do not reveal any statistically significant time trend.

Table 3 presents the result of the effect of safety net activities on hospital operating expenditure. In both the OLS and fixed effects specifications A higher SES index was associated with lower hospital expenditure and this effect was significant. For Medicaid intensity, the OLS model found that Medicaid intensity was associated with decreased operating expenditure. However, in the fixed effects specification, Medicaid intensity did not significantly impact operating expenditure indicating that the OLS results were due to time invariant hospital characteristics. Here also, we experimented with 2SLS. Results from Hausman specification test indicated that Medicaid admission was endogenous in the expenditure models. However, the second stage estimates were too imprecise to be informative, our standard errors for second stage increased from 0.02 to 0.25. The models that included uncompensated care burden did not affect the safety net and Medicaid intensity coefficients. In addition, there was no evidence of significant effect of uncompensated care burden on operating expenditure and its time interactions did not reveal any statistically significant time trend.

**Table 3:** Effect of safety net activities on operating expenditure

	**Continuous safety net measures**		**Binary safety net measures**
	
	**(1) OLS**	**(2) Fe**	**(3) Fe-Binary**
	
Log Medicaid intensity * 1990	-0.239 [0.074]**	-0.090 [0.053]	
Log Medicaid intensity * 1991	-0.245 [0.073]**	-0.115 [0.046]*	-0.007 [0.011]
Log Medicaid intensity * 1992	-0.225 [0.077]**	-0.077 [0.042]	-0.008 [0.013]
Log Medicaid intensity * 1993	-0.162 [0.076]*	-0.031 [0.038]	0.003 [0.014]
Log Medicaid intensity * 1994	-0.170 [0.077]*	-0.028 [0.037]	-0.003 [0.017]
Log Medicaid intensity * 1995	-0.043 [0.076]	-0.013 [0.038]	0.001 [0.019]
Log Medicaid intensity * 1996	0.139 [0.080]	0.035 [0.041]	0.011 [0.021]
Log Medicaid intensity * 1997	0.161 [0.078]*	0.026 [0.043]	0.010 [0.024]
Log Medicaid intensity * 1998	0.230 [0.075]**	0.062 [0.047]	0.011 [0.025]
Log Medicaid intensity * 1999	0.231 [0.070]**	0.013 [0.048]	0.006 [0.026]
SES index *1990	-0.008 [0.007]	-0.086 [0.014]**	
SES index *1991	-0.011 [0.007]	-0.087 [0.013]**	-0.005 [0.012]
SES index *1992	-0.005 [0.007]	-0.084 [0.013]**	0.015 [0.015]
SES index *1993	-0.012 [0.007]	-0.086 [0.013]**	0.008 [0.016]
SES index *1994	-0.012 [0.007]	-0.086 [0.013]**	0.026 [0.020]
SES index *1995	-0.017 [0.007]*	-0.086 [0.013]**	0.036 [0.023]
SES index *1996	-0.024 [0.007]**	-0.090 [0.013]**	0.033 [0.024]
SES index *1997	-0.028 [0.007]**	-0.094 [0.014]**	0.026 [0.026]
SES index *1998	-0.028 [0.007]**	-0.099 [0.014]**	0.029 [0.026]
SES index *1999	-0.033 [0.007]**	-0.102 [0.014]**	0.019 [0.027]
log(case mix index)	2.953 [0.471]**	0.054 [0.329]	0.156 [0.331]
log(discharges)	0.163 [0.137]	0.436 [0.128]**	0.453 [0.132]**
Log(visits)	0.271 [0.106]*	0.003 [0.053]	0.002 [0.054]
Log(discharges) squared	0.068 [0.010]**	0.028 [0.008]**	0.031 [0.008]**
Log(visits) squared	0.012 [0.007]	0.020 [0.003]**	0.022 [0.003]**
Log(case mix)*log(discharged)	-0.216 [0.051]**	0.024 [0.037]	0.014 [0.037]
Log(visits)*log(discharges)	-0.047 [0.014]**	-0.044 [0.007]**	-0.050 [0.008]**
Log(Medicare wage index)	0.371 [0.393]	0.216 [0.374]	0.267 [0.382]
Log(Medicare wage index)squared	0.106 [0.135]	-0.117 [0.091]	-0.109 [0.091]
Log(visits)*log(Medicare wage index)	0.132 [0.042]**	-0.031 [0.025]	-0.045 [0.026]
Log(discharges)*log(Medicare wage index)	-0.169 [0.049]**	0.015 [0.042]	0.027 [0.042]
Log(intern-to-bed ratio)	0.915 [0.049]**	0.174 [0.086]*	0.151 [0.086]
Not-for-profit	-0.005 [0.011]		
For-profit	-0.022 [0.014]		
1991 dummy	0.123 [0.022]**	0.137 [0.014]**	0.128 [0.011]**
1992 dummy	0.227 [0.028]**	0.249 [0.017]**	0.258 [0.014]**
1993 dummy	0.302 [0.033]**	0.328 [0.020]**	0.354 [0.017]**
1994 dummy	0.395 [0.035]**	0.396 [0.024]**	0.429 [0.020]**
1995 dummy	0.354 [0.038]**	0.434 [0.027]**	0.475 [0.022]**
1996 dummy	0.343 [0.041]**	0.480 [0.029]**	0.537 [0.024]**
1997 dummy	0.357 [0.043]**	0.544 [0.030]**	0.601 [0.026]**
1998 dummy	0.390 [0.045]**	0.596 [0.033]**	0.669 [0.028]**
1999 dummy	0.445 [0.045]**	0.696 [0.033]**	0.757 [0.029]**
Log percent Medicare*1990	0.104 [0.059]	0.367 [0.066]**	0.398 [0.066]**
Log percent Medicare*1991	0.059 [0.061]	0.306 [0.063]**	0.357 [0.063]**
Log percent Medicare*1992	0.054 [0.062]	0.280 [0.058]**	0.310 [0.059]**
Log percent Medicare*1993	0.034 [0.066]	0.261 [0.055]**	0.274 [0.056]**
Log percent Medicare*1994	-0.011 [0.063]	0.248 [0.054]**	0.260 [0.055]**
Log percent Medicare*1995	0.117 [0.063]	0.258 [0.053]**	0.269 [0.055]**
Log percent Medicare*1996	0.217 [0.068]**	0.290 [0.054]**	0.287 [0.055]**
Log percent Medicare*1997	0.275 [0.069]**	0.285 [0.055]**	0.290 [0.056]**
Log percent Medicare*1998	0.253 [0.070]**	0.270 [0.058]**	0.266 [0.060]**
Log percent Medicare*1999	0.186 [0.071]**	0.154 [0.060]**	0.175 [0.062]**
Log(System HHI)*1990	-0.135 [0.013]**	-0.135 [0.020]**	-0.172 [0.020]**
Log(System HHI)*1991	-0.125 [0.013]**	-0.128 [0.020]**	-0.161 [0.020]**
Log(System HHI)*1992	-0.100 [0.014]**	-0.109 [0.020]**	-0.138 [0.020]**
Log(System HHI)*1993	-0.078 [0.014]**	-0.094 [0.020]**	-0.120 [0.020]**
Log(System HHI)*1994	-0.048 [0.015]**	-0.074 [0.020]**	-0.094 [0.020]**
Log(System HHI)*1995	-0.045 [0.014]**	-0.065 [0.019]**	-0.082 [0.020]**
Log(System HHI)*1996	-0.041 [0.015]**	-0.058 [0.021]**	-0.071 [0.021]**
Log(System HHI)*1997	-0.030 [0.016]	-0.044 [0.021]*	-0.053 [0.022]*
Log(System HHI)*1998	-0.000 [0.016]	-0.024 [0.022]	-0.027 [0.022]
Log(System HHI)*1999	0.000 [0.017]	-0.023 [0.022]	-0.022 [0.022]
Mean HMO penetration*1990	0.047 [0.037]		
Mean HMO penetration*1991	-0.013 [0.037]	-0.037 [0.013]**	-0.035 [0.012]**
Mean HMO penetration*1992	-0.084 [0.036]*	-0.096 [0.018]**	-0.103 [0.016]**
Mean HMO penetration*1993	-0.113 [0.036]**	-0.125 [0.020]**	-0.136 [0.019]**
Mean HMO penetration*1994	-0.146 [0.037]**	-0.143 [0.025]**	-0.158 [0.023]**
Mean HMO penetration*1995	-0.166 [0.037]**	-0.154 [0.028]**	-0.174 [0.026]**
Mean HMO penetration*1996	-0.170 [0.036]**	-0.174 [0.031]**	-0.195 [0.030]**
Mean HMO penetration*1997	-0.150 [0.036]**	-0.178 [0.033]**	-0.199 [0.031]**
Mean HMO penetration*1998	-0.090 [0.038]*	-0.157 [0.035]**	-0.181 [0.034]**
Mean HMO penetration*1999	-0.086 [0.039]*	-0.199 [0.037]**	-0.220 [0.036]**
Observations	16680	16680	16680

Notes:

Source: AHA Annual Hospital Survey and Medicare Hospital Cost Reports (1990-1999)

^a^A high score on SES index indicates low socioeconomic status Robust standard errors in brackets

*Significant at p < 0.05; ** significant at p < 0.01%

## Discussion

Our study found that after controlling for the major hospital, market, and policy variables, safety net activities had relatively small, and in general statistically insignificant, effects on hospital profit margins throughout the 1990s. Medicaid intensity, in both OLS and fixed effects specifications had little effect on profit margin. From the regression models, hospitals with high Medicaid intensities (highest 5^th ^percentile) had somewhat higher profit margins, significantly so after 1994. In the OLS specifications, low-SES population was associated with reduced profit margin and the magnitude of this effect increased over time but was still relatively small. This relationship, however, disappeared in both the fixed effects specification and for hospitals serving the lowest SES (highest SES index) populations. In summary, during the period studied, safety net activity had only a small effect on hospital profitability.

Our second objective was to assess the effect of safety net activities on operating expenditure. Had hospitals responded by maintaining profitability at the cost of decreasing their quality of care? The hospitals can reduce the quality of the care by either altering the mix of services or number of services provided to the uninsured. Our expenditure model indicated that during our study period there was a negative relationship between the SES index and hospital expenditures. We did not find evidence of a similar relationship with Medicaid intensity. Hospitals serving lower SES (higher SES index) populations had significantly lower expenditures compared to hospitals with high SES populations. In models with continuous SES index this effect remained consistently negative for the entire study period. Interestingly, in the models with "binary" variables, there was no significant difference in expenditures. Medicaid intensity was negatively related to expenditures from 1990 to 1995 but only reached statistical significance in 1991. After 1995 the relationship was positive but not significant. There was no statistically significant effect of Medicaid intensity in these models. Although the uncompensated care data were only available for 1 year, there was no evidence of negative effect of uncompensated care burden on profit margin and operating expenditure. Further, the addition of this variable to the models did not significantly affect the coefficients of Medicaid intensity and SES index.

### Study limitations

Representativeness-Neither the Medicare Hospital Cost Report nor the American Hospital Association reports contains usable data for all urban hospitals. The use of a sub-set of hospitals in an analysis raises concerns for the representativeness regarding our sample. This concern is mitigated for the primary analyses both by the large proportion of all urban hospitals in our sample and by the fact that the mean differences between key hospital characteristics (e.g., ownership, Medicaid admission, and size) were small across observations that were included in the analysis and those that were omitted due to missing data.

#### Time period studied

The BBA was enacted in 1997 and had not yet had its full impact by 1999. It is unclear whether the relationships observed in the 1990s will persist after 1999 as greater cuts are phased in. The observed trend, towards lower expenditures in the later years, does suggest that the BBA may have had a significant impact, especially with higher Medicaid intensity being associated to lower expenditures. Additional years of data will be required to enable a test of the stability of these relationships.

#### Definition of safety net activities

Prior studies of safety net hospitals have also used public ownership and/or teaching status or the disproportionate provision of uncompensated care to designate safety net hospitals. Teaching status and ownership were included as covariates in the OLS specifications. In general, our results paralleled those seen in prior studies, teaching hospitals tended to have lower profits and higher expenditures, public hospitals tended to have lower profits and similar expenditures independent of the level of their safety net activities. A more serious concern arises from the omission of uncompensated care from the primary analyses. The provision of uncompensated care is clearly a critical safety net dimension, and the uncompensated care burden should be included in an analysis of the effects of safety net activities. We excluded this measure from the primary analyses simply because of the limited data that were available at the time of the analysis. The CMS Hospital Cost Reports-responding to a pressing public policy need-began to collect uncompensated care data for the 2002-2003 period. We included these data in some test specifications, based on the observation that the hospitals' provision of uncompensated care is relatively stable-and so highly correlated from year to year. The inclusion of this variable had almost no effect on the coefficients of the other two variables, and it had a weak and statistically insignificant relationship with both dependent variables. Analysis of data from a later study period should be performed to confirm these findings.

We also acknowledge the conceptual limitations associated with our safety net activities. For instance, our safety net measures assume that Medicare service area is similar to hospitals commercial and Medicaid service area. Ideally, we would have liked to use a validated national discharge data; however, data availability precludes that. More importantly, prior work by Goodman et al. [38] indicates that the services area for Medicaid, Medicare, and commercial population were similar.

#### Association rather than causality

This study attempts to assess whether increases in safety net activities lower total profitability and/or operating expenditure. Causality is notoriously difficult to prove in the social sciences, and this study is no exception. Given that regression analyses measure the degree of association between the dependent and independent variables, one can only infer the plausibility of a causal relationship. In addition to these general concerns, there is a specific concern because one might expect Medicaid intensity to be endogenous. With regard to overall causality, we used OLS and FE specifications since the two test for different dimensions of the relationship between dependent and independent variables-the former, the overall association; the latter relates changes over time in the dependent and independent variables for each hospital. In most cases, the two specifications are consistent, strengthening the plausibility of the relationship. The OLS results indicate that a higher SES index reduces profitability, whereas the fixed effects specification shows no statistically significant relationship. These findings are broadly consistent with the aggregate data-hospitals in lower SES areas were less profitable at any given point in time, but their trends over time were no different than the other hospitals. In addition, the non-significant relationship in the fixed effects models may reflect the fact that the SES index only has small variation over time since it is anchored on three points (1990, 1995, and 2000). To the extent possible, we tested for endogeneity and found that the results of our analysis are consistent with the exogeneity of Medicaid admissions for the profit margin model, even if these results are not definitive. These results suggest that the observed relationships are robust.

### Implications

The results of our study suggest that the political and market turbulence during the 1990s did not disproportionately impact hospitals serving vulnerable populations and/or having high Medicaid intensities. For the most part, these increased safety net activities, whether measured continuously or for the highest 5^th ^percentile, did not negatively affect financial performance to a significant extent during the study period. Even when these relationships did reach statistical significance, their effects were small. It is striking that the two safety net dimensions that are recognized in public policy, the disproportionate provision of services to Medicaid beneficiaries and uncompensated care (to the extent possible given the data limitations), do not appear to be associated with any reduction in operating expenditure. It is the third safety net dimension, serving a low-SES population, which is associated with a reduction in profitability and operating expenditure possibly because it is not used explicitly to allocate subsidies, although even here the impacts are relatively small. These results suggest that hospitals providing these activities are unlikely to face widespread closures. So does this mean that hospitals serving poor don't need public support? On the contrary, because our dependent variable includes government subsidies and because figure 1 indicates that operating margins were negative, the results signify the importance of government support in maintaining financial viability. The methods used by safety net hospitals to maintain financial viability are beyond the scope of this study but Felland et al. (2003) provide some possible explanation. The results of their study indicated that safety net hospitals have been able to expand and improve services to the uninsured by streamlining their operations, engaging in integration, and actively pursuing paying patients.

There are some more disquieting suggestions. On average, hospitals with vulnerable population profit margins might have followed overall trends, but this could have occurred at the expense of lower quality for hospitals with low-SES populations. Furthermore, some of the time trends for the late 1990s suggest increasing financial pressure on hospitals serving vulnerable populations. Although the impacts may not have been disproportionate, since such hospitals often started from lower profitability, these trends could indicate more financial pressure than for hospitals beginning with greater financial cushions. These results taken together suggests that hospitals serving vulnerable populations were successful in responding to financial pressure, but the effects of these responses on patient's outcomes needs to be evaluated.

## Conclusions

In summary, the complex system of direct and indirect subsidies provided to hospitals during the 1990s appears to have been relatively successful in compensating them for the costs of their safety net activities. However, these analyses need to be extended into the period where the full impact of the BBA was felt in order to provide a more definitive portrait of the financial health of hospitals serving the most vulnerable populations.

## List of abbreviations used

(BBA): Balanced Budget Act; (CMS): Centers for Medicare and Medicaid Services; (HMO): Health Maintenance Organization; (HHI): Hirschman-Herfindal indices; (HMAF): Hospital Market Service, Area Files; (MSA): Metropolitan statistical area; (OLS): Ordinary least square; (SES): Socioeconomic status; (ZCA): Zip code area

## Competing interests

The authors declare that they have no competing interests.

## Authors' contributions

JZ designed the study, conceptual framework, and drafted the manuscript. NK participated in the design of the study, conducted data analysis, and drafted the manuscript. AB participated in data analysis, and assisted in drafting of the manuscript.

## Pre-publication history

The pre-publication history for this paper can be accessed here:

http://www.biomedcentral.com/1472-6963/10/15/prepub
